# Semiochemical mediated enhancement of males to complement sterile insect technique in management of the tephritid pest *Bactrocera tryoni* (Froggatt)

**DOI:** 10.1038/s41598-017-13843-w

**Published:** 2017-10-17

**Authors:** Mohammed Abul Monjur Khan, Nicholas C. Manoukis, Terry Osborne, Idris M. Barchia, Geoff M. Gurr, Olivia L. Reynolds

**Affiliations:** 1 Biosecurity and Food Safety, NSW Department of Primary Industries, Elizabeth Macarthur Agricultural Institute, Menangle, New South Wales, Australia; 2 Department of Entomology, Faculty of Agriculture, Bangladesh Agricultural University, Mymensingh, 2202 Bangladesh; 3 USDA-Agricultural Research Service, Daniel K. Inouye US Pacific Basin Agricultural Research Center, Hilo, Hawaii 96720 USA; 4 Graham Centre for Agricultural Innovation (an alliance between NSW Department of Primary Industries and Charles Sturt University), Locked Bag 588, WaggaWagga, NSW 2678 Australia; 5 State Key Laboratory for Ecological Pest Control of Fujian and Taiwan Crops, Fujian Agriculture and Forestry University, Fuzhou, 350002 China

## Abstract

Queensland fruit fly, *Bactrocera tryoni* (Froggatt), is the most significant pest of Australia’s $9 billion horticulture industry. The sterile insect technique (SIT) and cue-lure (a synthetic analogue of raspberry ketone (RK))-based male annihilation technique (MAT) are two of the most effective management tools against this pest. However, combining these two approaches is considered incompatible as MAT kills sterile and ‘wild’ males indiscriminately. In the present study we tested the effect of pre-release feeding of *B. tryoni* on RK on their post-release survival and response to MAT in field cages and in a commercial orchard. In both settings, survival was higher for RK supplemented adults compared to control (i.e. RK denied) adults. A lower number of RK supplemented sterile males were recaptured in MAT baited traps in both the field cages and orchard trials compared to RK denied sterile males. The advantage of this novel “male replacement” approach (relatively selective mortality of wild males at lure-baited traps while simultaneously releasing sterile males) is increasing the ratio of sterile to wild males in the field population, with potential for reducing the number of sterile males to be released.

## Introduction

Fruit flies of economic importance belong to the family Tephritidae, which is among the largest families of Diptera, comprising approximately 4000 species^1^. A total of 180 fruit fly species are established as economically significant pests across 118 countries^2^. The most important fruit fly pest species in Australia is the Queensland fruit fly, *Bactrocera tryoni* (Froggatt), which attacks almost all commercial fruits and many vegetables^3^. *Bactrocera tryoni* causes economic losses through direct crop damage and associated control costs and is a significant barrier to national and international horticultural market access^4^. Among management options for this pest, the sterile insect technique (SIT) and male annihilation technique (MAT) are two important Area Wide Integrated Pest Management tools currently in use in Australia at national, regional and smaller scales^4^.

In SIT programs, sterile males are released to mate with wild females; the mated females are then unable to produce viable offspring^5,6^. Consequently, pest populations are suppressed or may even be eradicated^5^. In SIT programs, tephritids, including *B. tryoni*, are typically released as immature adults, one to three days after eclosion^7–9^. Therefore, upon release, sterile males must first survive to maturation before successfully locating and copulating with wild females^10^.

SIT programs are more effective and less costly if the density of wild individuals is reduced prior to the release of sterile insects, because this reduces the numbers of sterile insects that need to be reared and released. For tephritids, MAT is a potential means of reducing populations of wild males^11,12^. MAT uses large numbers of ‘bait stations’, containing a male attractant and an insecticide, which can significantly reduce local male densities^11,13–15^. Bait stations may take multiple forms, with some recent methods employing a waxy matrix that contains an attractant and insecticide^11,15–18^.

For managing *B. tryoni*, MAT and SIT are typically used consecutively rather than concurrently, because MAT kills sterile as well as wild males. Accordingly, MAT is used as a first step to reduce local densities of wild males. Thereafter, MAT is typically terminated, and sterile males released. Modelling studies^19^of tephritids, other than *B. tryoni*, suggest that MAT and SIT are synergistic and are more effective in combination than when used alone or consecutively provided that traps preferentially capture wild rather than sterile males.

Studies of several *Bactrocera* species show that pre-release exposure of males to plant-derived semiochemicals and synthetic lures dramatically reduces their subsequent response to attractant lures used in MAT^20,21^. Several commercially available lures and other male attractants are known for *B. tryoni*, including cue-lure, melolure, raspberry ketone (RK), RK formate, RK trifluoroacetate, and zingerone, which are all structurally similar^11,15,22–25^. While RK and zingerone are naturally occurring, cue-lure, RK formate, and RK trifluoroacetate are synthetic analogues of RK^26^. However, RK and cue-lure are more attractive than zingerone to *B. tryoni* ^27^. Usually, the majority of *B. tryoni* are attracted to cue-lure when mature, i.e. 8–12 days age^28^. Recent laboratory studies have shown that feeding by fertile *B. tryoni* males on cue-lure and RK triggers physiological changes that lead to accelerated sexual maturation and enhanced mating success^29,30^, but the consequent effect on male response to MAT devices has not been studied. Further, studies to date have used fertile males and have not considered sterile males as used in SIT.

Here, we consider the hypothesis that pre-release feeding on RK by immature sterile adult male *B. tryoni* may change their subsequent response to cue-lure based MAT. This is important because a reduction in attraction of sterile males would open the practical possibility of using MAT concurrently with SIT. Accordingly, this study examined the response of sterile *B. tryoni* to cue-lure in field cages and in a commercial orchard after feeding on RK prior to release. Subsequent survival of sterile male *B. tryoni* was also assessed to examine any adverse effects of pre-release RK exposure.

## Results

### RK supplementation improves *B. tryoni* survival

#### Field cage assay

**Figure 1:**
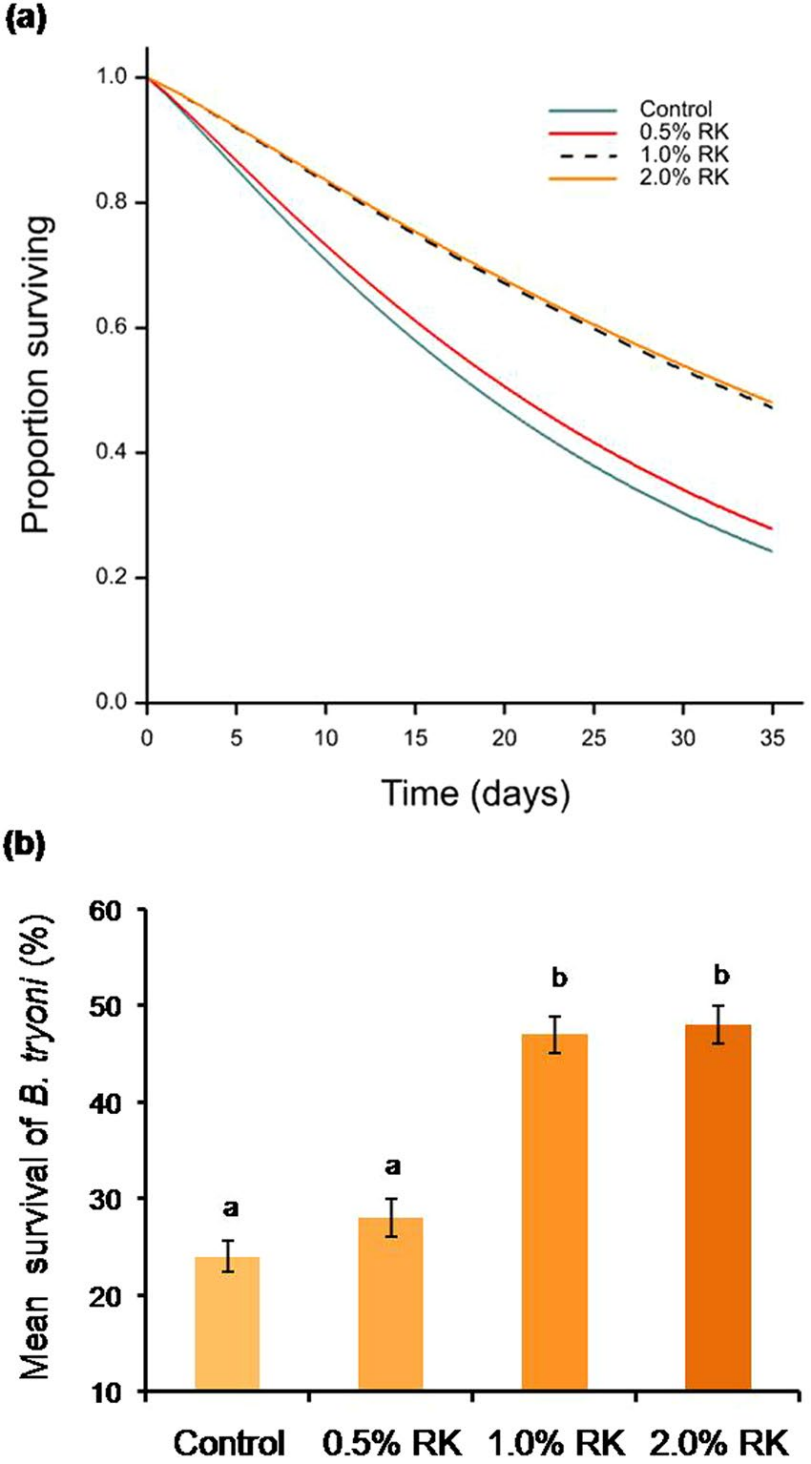
Survival of sterile male *B. tryoni* when fed a raspberry ketone (RK) supplemented diet when immature. (**a**) Survivorship curves show the daily survival (**b**) mean survival after 5 weeks. Means followed by the same letter are not significantly different (p > 0.05).

Mortality of sterile male *B. tryoni* was reduced when they were fed on a diet of 1% or 2% RK compared with the control (RK denied). The shape of the survival curves differed between the four RK treatments (F_4,545_ = 8.03; p < 0.001) (Fig. 1a). No significant difference was found in the daily mortality rate between control and 0.5% RK fed *B. tryoni* (F_2,247_ = 0.24; p > 0.05; Fig. 1a). The daily mortality rate for each RK diet treatment was 2.57% (0%), 2.32% (0.5%), 1.36% (1%) and 1.33% (2%). The mean survival of sterile male *B. tryoni* after 5 weeks was also higher for 1% and 2% RK diet treatments than 0.5% or the control (p < 0.05; Fig. 1b).

#### Commercial orchard assay

The survival of RK supplemented *B. tryoni* was higher compared to the control. The estimated daily survivorship of RK supplemented *B. tryoni* was 0.9407 (dependent variable: *ln*[1 + mean recaptures] independent variable: time since last release; intercept = 1.7943; slope = −0.06113). This is equivalent to survival of 11.7% at 5 weeks. For control *B. tryoni*, daily survivorship was estimated to be 0.9337 using the same linear regression model (intercept = 2.1903; slope = −0.0687), equating to 9.04% survival at 5 weeks. Full regression details are given in Table 1.

**Table 1:** Effect of pre-release feeding raspberry ketone to immature sterile male *B. tryoni* on survival in a commercial orchard.

Source	Value	SE	*t*	*p*
**RK-fed** ***B. tryoni***				
Intercept	1.794	0.440	4.075	0.027
Time since last release	−0.061	0.019	−3.223	0.049
Residual SE 0.420 (3 df); adjusted R^2^ 0.701; model F^2^ 10.39; *p* 0.048				
**Control** ***B. tryoni***				
Intercept	2.190	0.427	5.130	0.014
Time since last release	−0.069	0.018	−3.733	0.034
Residual SE 0.407 (3 df); adjusted R^2^ 0.764; model F^2^ 13.94; *p* 0.034				

Linear regression model of log(1 + average recapture of RK fed flies or RK-denied flies) as predicted by time since last release (in days). Standard error (SE), t-statistics (*t*), and p-values (*p*) are shown.

### RK supplementation reduces the attraction of sterile male *B. tryoni* to cue-lure

#### Field cage assay

**Figure 2:**
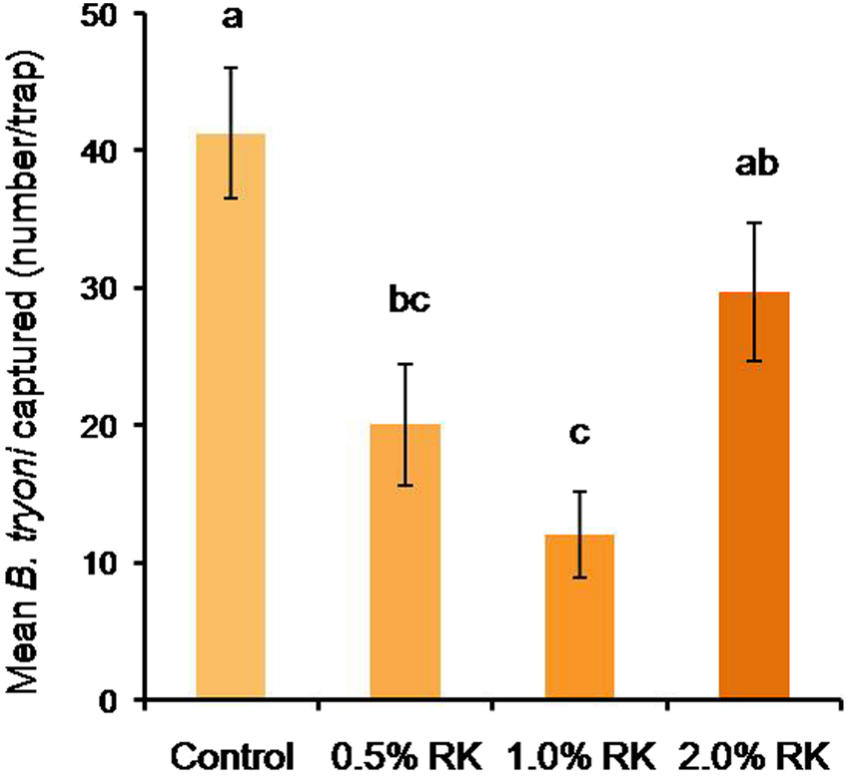
Effect of feeding raspberry ketone (RK) to immature sterile male *B. tryoni* on their recapture in cue-lure baited Lynfield traps in walk-in field cages. Means followed by the same letter are not significantly different (p > 0.05).

The number of RK supplemented and control sterile males recaptured in cue-lure baited traps differed significantly among treatments (F_3,10_ = 8.96; p = 0.003; Fig. 2). Reduced numbers of 0.5% and 1% RK supplemented sterile male *B. tryoni* were captured compared to the RK denied control males (F_1,10_ = 21.92; p < 0.001). Sterile male recaptures did not differ between 2% RK and control males (F_1,10_ = 2.71; p = 0.131; Fig. 2).

#### Commercial orchard assay

Pre-release RK supplementation of the immature fly diet changed the response of sterile male *B. tryoni* to cue-lure in field trials. A significantly lower number of RK fed individuals were found in cue-lure baited traps in the orchard compared to RK denied control flies (p = 0.01; Table 2).

**Table 2:** Mean number of sterile male *B. tryoni* trapped per week, when supplemented pre-release as immatures (2-3 days) with raspberry ketone (RK), or denied RK (i.e. control) across a commercial orchard.

Treatment	Mean sterile male *B. tryoni*/trap/week		
	Means	Lower limit	Upper limit
Control	0.661 a	0.591	0.740
RK-fed	0.486 b	0.426	0.554

Mean value followed by the differing letters are significantly different from one another (p > 0.05).

## Discussion

Here we show that simultaneous use of SIT and MAT is made viable by pre-release feeding of sterile *B. tryoni*. RK treatment increased the survival of sterile *B. tryoni* males under semi-field experimental conditions, with validation under commercial field conditions. In a SIT program, millions of sterile flies are typically released in the field, so even a slight improvement in the survival rate will result in increased abundance of sterile *B. tryoni* and a higher ratio of sterile to wild males, leading to enhanced suppression of *B. tryoni* pest populations^4^.

While this is the first study to test immature sterile male *B. tryoni* under field conditions, two prior studies have investigated effects on fertile males in the laboratory. With continuous access to liquid cue-lure (>95% purity) over the first 8 weeks of adult life, *B. tryoni* males displayed greater mortality than control males denied access to cue-lure^29^. In contrast, a powdered form of RK (1.25–5%) mixed with the adult diet and provided to fertile flies aged 0–24 h, which were permitted to feed for 48 h, did not affect the longevity of *B. tryoni* ^30^. The inconsistency between those two studies is likely due to the markedly different lengths of feeding permitted, with only the 8-week long regime having an effect in the form of reduced survival. Reasons for the improved survival of *B. tryoni* in the present study are not clear but maybe associated with the RK supplementation technique.

There is now solid evidence that when sterile^31^ or fertile^32–34^ fruit fly males ingest a semiochemical lure, or its analogue, subsequent attraction to that same lure significantly decreases. The present study is, however, the first to show a reduced response to cue-lure of RK supplemented immature *B. tryoni*. Previous studies have focused on methyl eugenol-responding species, and to a lesser extent RK/cue-lure responding species^21,35^. *Bactrocera cucurbitae* was found to be 3–10 times less responsive to traps baited with cue-lure after 4-5 days exposure to cue-lure^32^. Other *Bactrocera* species, such as *B*. *dorsalis*, were less likely to be captured in methyl eugenol (ME) baited traps after being fed ME supplemented diets^33,34,36^. While two previous studies have demonstrated an effect of lure feeding with cue-lure and RK on *B. tryoni* mating performance^29,30^, the field cage study and the open field experiment results presented in this study support an additional advantage of reduced responsiveness to cue-lure by RK-supplemented immature *B. tryoni*.

In this study trap-capture of sterile *B. tryoni* was reduced with RK supplementation under field-cage and field conditions, although the response was variable. The higher RK treatment (2%) in the field cage assay resulted in higher captures relative to the 1% dose, and survival at the lowest tested dose (0.5%) was comparable to the control. However, the field study demonstrated increased survival and reduced trap capture of RK supplemented flies, when provided a low dose. Future studies are required to determine the optimal RK dose that provides both a reduced response to MAT and a positive (or neutral) influence on other physiological, ecological and behavioural traits of sterile males. As current *B. tryoni* SIT involves the release of sterile females in addition to males (i.e. bisex strain), the effects of RK on female longevity also requires investigation. Future field studies also need to use a survival monitoring method that does not employ a semiochemical lure to tease apart the effects on survival and attraction to lure-baited traps.

**Figure 3:**
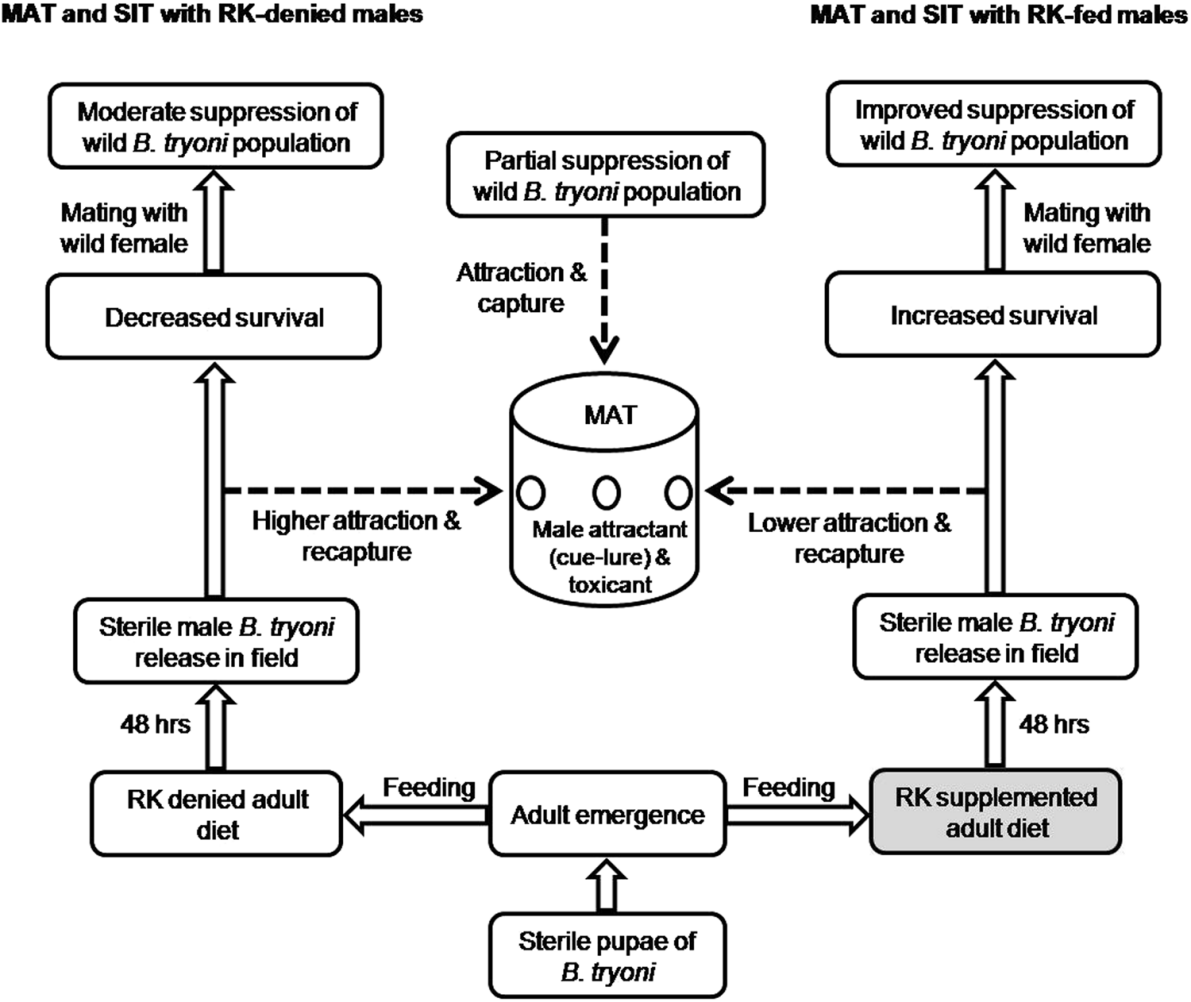
The comparative advantages of pre-release raspberry ketone (RK) supplementation of the immature sterile adult *Bactrocera tryoni* diet in sterile insect technique (SIT) programs which incorporate the use of the male annihilation technique (MAT), resulting in increased sterile male survival and a reduced response to MAT, and therefore increased suppression of wild *B. tryoni* populations.

The concept of the concurrent use of SIT and MAT to improve management of tephritids dates back to the 1970s, but its use has been hampered by the attraction of sterile as well as fertile male flies to MAT traps. Reducing captures of sterile flies, as the present study shows to be possible, is likely to allow increased SIT efficiency and reduced cost. Area wide management programs incorporating concurrent use of SIT and MAT would benefit from increased survival and reduced vulnerability to MAT by RK fed immature *B. tryoni* (Fig. 3) and may reduce the number of sterile flies required for release.

## Conclusion

Area wide management SIT programs are costly, and reducing the cost is a continual focus of research efforts. This study shows the first evidence that a diet containing RK fed to immature sterile *B. tryoni* prior to release improves subsequent survival of sterile males and reduces their response to MAT. Semiochemical-mediated enhancement of sterile flies, utilising RK, has the potential to enable simultaneous use of SIT and MAT in operational SIT programs, reducing costs and increasing effectiveness of such programs.

## Methods

### Study Insects

*Bactrocera tryoni* (bisex strain) were obtained as pupae from the Fruit Fly Production Facility (FFPF) at the Elizabeth Macarthur Agricultural Institute, Menangle, New South Wales (NSW), Australia. Insects were reared on a standard lucerne chaff diet^37,38^ in a controlled environment room under standard conditions (i.e., 26 ± 2 °C, 65 ± 10% relative humidity [RH], and a photoperiod of 14:10 [L:D] h, with a simulated dawn and dusk as the lights ramp up and down at the beginning and end of the light phase). Late-stage *B. tryoni* pupae were irradiated under hypoxia at 60–65 Gy of gamma irradiation from a cobalt-60 source at the Australian Nuclear Science and Technology Organisation (ANSTO) facility at Lucas Heights, NSW, which is the standard protocol to produce insects for SIT in Australia.

### Effect of RK supplementation on the survival of *B. tryoni*

#### Field cage assays

Each week, approximately 10 g of sterile pupae (approximately 1000 flies) were placed in each of four cages (30 × 30 × 30 cm, Bugdorm cage, Megaview Science Co Ltd, Taiwan) and maintained during the experimental period under standard laboratory conditions. Soon after adult eclosion (0–24 h), remaining unemerged pupae were discarded, and the newly emerged adults (age 0–24 h) were provided with 100 g of a single RK diet treatment (see below) on a Petri dish and water and permitted to feed for 48 h. Prior to sterile male release in field cages (see below), females were separated and discarded.

The agar based diet for adult *B. tryoni* was prepared with the following ingredients: agar, sugar and yeast hydrolysate enzymatic (MP Biomedical, LLC, OH, USA; 60% protein) (3:1 by weight), and water. To prepare the diet, sugar and agar were mixed with water and boiled for 5–10 min. The mixture was allowed to cool to 40 °C, then yeast hydrolysate enzymatic was added to this mixture and stirred gently until combined.

The RK powder (99%, Sigma Aldrich, USA) was first dissolved in absolute ethanol and left for 24 h prior to incorporation with the agar-based diet. Immediately after diet preparation, the RK solution was added to the diets separately at a concentration of 0.0% (control), 0.5%, 1.0%, and 2.0% RK and then gently stirred with an electric stirrer to achieve uniform mixing. The diet was then allowed to cool and solidify for 30 min at room temperature before use.

To evaluate the survival of sterile male flies in field cages, PVC pipe-framed field cages (1.5 × 1 × 1 m) with zippers to allow access into the cage were placed over potted citrus trees (*Citrus aurantium*, Seville Orange) that had leaves but no fruit or flowers. Fifty sterile male flies, aged 48–72 h from each RK diet treatment were released in a field cage, and a total of three cages were maintained for each treatment. Each field cage contained yeast hydrolysate and sugar as food and water provided *ad libitum*. The number of dead flies were recorded daily and removed from the field cages. The trial continued until over 75% fly mortality was reached in the control treatment at five weeks after fly release. Mean climatic conditions during the survival assay periods were 21.95 ± 0.10 °C (min), 28.0 ± 0.40 °C (max) and 19.1 ± 1.18% RH (min), 58.0 ± 5.75% RH (max).

#### Commercial orchard Study site

Warroo (27°38′00″S, 148°45′00″E) near Stanthorpe, Queensland is a 60 ha orchard (approximately 75,000 trees) of plum variety ‘Queen Garnet’ and pollinator trees. Twenty sterile fly release sites on a 400 m grid were established. Release sites were located central to four trapping sites, located at least 150 m from any trap.

#### Sterile adult release protocol

Irradiated pupae obtained from the FFPF as described above were transported by air and road to Warroo. Upon receipt, pupae were divided into 200 g lots and placed inside translucent lidded plastic adult rearing containers (PARCs) (Silverlock MH 0110, colour ‘natural’, size: 645 × 413 × 275 mm; or Ezy Storage 31619, colour ‘clear’, size: 570 × 354 × 380 mm), with a 430 × 200 mm, mesh on the lid and a 150 × 100 mm mesh on two sides of the container for ventilation. Additional resting space was provided by wedging cardboard dividers (approximately 160 mm in height) inside each container. To distinguish between treatments and release periods, the pupae were marked with 0.8 g dye (Fiesta FEX 1 fluorescent pigments, Swada, 30–32 Kilkenny Court, Dandenong South, Victoria, Australia) per 100 g pupae, with dye colour (arc chrome, strong magenta, pink and stellar green) alternated between treatment types for each period of release. Dye pigments used in this experiment have no significant effect on emergence, flight and trap recapture rates for *B. tryoni* ^39^.

Two release periods, each comprising weekly releases over five consecutive weeks, were conducted, and average pupal weights ranged between 9.0–9.9 mg for release period 1 and between 9.2–9.7 mg for release period 2, which was within the expected range produced by the FFPF^38^.

During each release, PARCs containing sterile flies were taken to the release sites, and one PARC containing RK-fed flies and one PARC containing RK-deprived flies was opened at each site and the flies allowed to disperse freely. The sterile *B. tryoni* were aged 3 days when released.

#### Diet treatments

For flies fed 0.2% RK, an agar block (6 × 4 × 2 cm) containing a mixture of white sugar, yeast hydrolysate (3:1 by weight) and water^40^, mixed with RK was placed inside the PARC (20 PARCs) on a plastic board on top of cardboard dividers before fly emergence. For ‘RK-deprived’ flies, an agar block with yeast hydrolysate and sugar was provided without the added RK (a further 20 PARCs). The pupae in the PARCs were acclimated under field conditions in a roofed shed until release. Mean climatic conditions were for release period 1: 14.4 ± 0.51 °C (min), 26.0 ± 0.42 °C (max) and 54.0 ± 3.14% RH (min), 74.4 ± 1.53% RH (max) and release period 2: 10.2 ± 0.58 °C (min), 22.8 ± 0.36 °C (max) and 49.1 ± 2.21% RH (min), 72.7 ± 1.8% RH (max). Temperature and RH data for Warroo, Queensland, were obtained from the Bureau of Meteorology, Australia.

#### Recapture of sterile male B. tryoni

Recaptures were made using a 400 m spaced trapping grid comprising 21 Lynfield traps baited with cue-lure (International Pheromones, London) and malathion^41^ positioned at 1.5–2 m height in trees. Traps were located throughout the orchard, in adjacent native vegetation, and two single residential properties, each located within several hundred metres from the orchard. Traps were cleared weekly after each release until six weeks after the final release. Vials containing trapped flies were then sent to EMAI, where they were assessed and classified by colour as RK-fed or RK-denied^39^. The total number of *B. tryoni* (for each RK diet treatment) caught in the trapping grid was used to estimate survival and response to cue-lure baited traps.

### Effect of RK supplementation on the attraction of sterile male *B. tryoni* to cue-lure

#### Field cage assay

After 48 h, sterile male *B. tryoni*, from each RK diet treatment were transferred and maintained in individual BugDorm cages (120 flies/cage). Food and water were provided as described above until release in field cages.

To evaluate the attraction of mature male flies to MAT lures, a single potted citrus tree (as described above) was placed in each of four walk-in circular field cages (4.0 m diameter, 2.5 m height). In each cage, a single Lynfield trap, baited with a MAT comprising cue-lure and malathion (Bio Trap Australia Pty Ltd), was placed in the tree canopy at 1.5 m height. One hundred sterile male flies aged 10 days from a single diet treatment were released in a field cage. The number of flies recaptured in traps was recorded every 24 h for 4 consecutive days. The experiment was repeated four times using sterile *B. tryoni* from four different generations, with a total of 1600 flies tested for their responsiveness to cue-lure. Mean environmental conditions during the periods of MAT lure response evaluation were 16.81 ± 0.89 °C (min), 35.73 ± 1.28 °C (max) and 37.73 ± 3.08% RH (min), 85.99 ± 2.69% RH (max).

#### Commercial orchard

The protocol was as described above for survival.

### Statistical analyses

#### Effect of RK supplementation on survival of sterile male B. tryoni in field cages

Prior to survival analysis of data from field cages, observed time of an individual insect death was randomly allocated to time between the last observation time and the recorded time. This individual mortality time is also called accelerated failure time (AFT). The AFT were analysed using a survival analysis. After examination of the observed versus fitted survival function on iterated log (time), Weibull distribution was the best fit to the AFT.

The survival (S) function considered to fit the data (***t***) was as follows

$$S({\boldsymbol{t}})=\exp (-\lambda \,{{\boldsymbol{t}}}^{\alpha })$$

where $$\lambda =\exp (\sum {b}_{i}{X}_{i})$$;

X_i_ is the i^th^ treatment; *b*_i_ is the shape parameter of the i^th^ treatment, *α* is the scale parameter of Weibull distribution. The parameter *λ* measures the instantaneous mortality rate (i.e. daily mortality rate)^42^. All the parameters were estimated using a maximum likelihood estimation analysis, which was run via GenStat (Eighteenth Edition) for Windows using RSURVIVAL procedure. All significant differences are based on comparing the shape parameter (*b*_i_) estimates and their standard errors.

#### Effect of RK supplementation on the response of sterile male B. tryoni to cue-lure in field cages

To measure the responsiveness of *B. tryoni* to cue-lure in walk-in field cages, data on the number of males captured in cue-lure baited traps were analysed by fitting a generalized linear model with error assumed to follow a binomial distribution^43^. A logit link was used to relate the recapture data to the treatment effects and a restricted maximum likelihood (REML) analysis was used to estimate the effects. Least significant difference (LSD) at 5% level was used to determine significance between treatment effects.

#### Effect of RK supplementation on the survival and response of sterile male B. tryoni to cue-lure in a commercial orchard

The following model was used to relate recaptures of marked sterile males in traps baited with cue-lure to daily survival^44,45^:

$$A=Na{p}^{n}$$

where *A* is the number recaptured, *N* is the total number marked and released, *a* is the recapture probability, *p* is the daily survival probability and *n* is the number of days since release^44^. A least-squares linear regression of time since release against the natural logarithm of (1 + *A*) usually provides a good fit, and the antilogarithm of the slope can be interpreted to be *p*, the daily probability of survival. Survival at day *x* was calculated as *p* ^*x*^ ^46^. Overlapping releases occurred weekly for 5 weeks in Warroo. For analysis of the data, average per trap recaptures following the last release was considered. This was necessary due to overlapping sexual maturation and mortality of each released cohort, which could cause the number of responsive (“capturable”) flies to increase after release. As analysed, the recaptures are of a pool of flies released over those weeks, with average weekly per trap recaptures for the first 35 days since the last overlapping release as the response variable and time since last release in days as the independent variable. Only release period 1 was analysed as the model fit to the second period was not significant.

To analyse the responsiveness of the released flies to cue-lure in the field, a generalized linear model with errors assumed to follow a Poisson distribution with variance adjusted for heterogeneity was employed. A log-link function was used to relate insect counts to RK effects. Group means were compared using the least significant difference (LSD) test at 5% significant level. Observations within the first 3 weeks trapping dates were discarded from analysis due to very low trap catches.
